# Depression, Anxiety and Stress during COVID-19: Associations with Changes in Physical Activity, Sleep, Tobacco and Alcohol Use in Australian Adults

**DOI:** 10.3390/ijerph17114065

**Published:** 2020-06-07

**Authors:** Robert Stanton, Quyen G. To, Saman Khalesi, Susan L. Williams, Stephanie J. Alley, Tanya L. Thwaite, Andrew S. Fenning, Corneel Vandelanotte

**Affiliations:** 1 Cluster for Resilience and Well-being, Appleton Institute, Central Queensland University, 44 Greenhill Road, Wayville 5043, Australia; 2 School of Health, Medical and Applied Sciences, Central Queensland University, Bruce Highway, Rockhampton, Queensland 4702, Australia; q.to@cqu.edu.au (Q.G.T.); s.khalesi@cqu.edu.au (S.K.); s.p.williams@cqu.edu.au (S.L.W.); s.alley@cqu.edu.au (S.J.A.); t.thwaite@cqu.edu.au (T.L.T.); a.fenning@cqu.edu.au (A.S.F.); c.vandelanotte@cqu.edu.au (C.V.); 3 Physical Activity Research Group, Appleton Institute, Central Queensland University, 44 Greenhill Road, Wayville 504, Australia

**Keywords:** psychological distress, COVID, health behaviors, physical activity, smoking, alcohol intake

## Abstract

The novel coronavirus (COVID-19) has enforced dramatic changes to daily living including economic and health impacts. Evidence for the impact of these changes on our physical and mental health and health behaviors is limited. We examined the associations between psychological distress and changes in selected health behaviors since the onset of COVID-19 in Australia. An online survey was distributed in April 2020 and included measures of depression, anxiety, stress, physical activity, sleep, alcohol intake and cigarette smoking. The survey was completed by 1491 adults (mean age 50.5 ± 14.9 years, 67% female). Negative change was reported for physical activity (48.9%), sleep (40.7%), alcohol (26.6%) and smoking (6.9%) since the onset of the COVID-19 pandemic. Significantly higher scores in one or more psychological distress states were found for females, and those not in a relationship, in the lowest income category, aged 18–45 years, or with a chronic illness. Negative changes in physical activity, sleep, smoking and alcohol intake were associated with higher depression, anxiety and stress symptoms. Health-promotion strategies directed at adopting or maintaining positive health-related behaviors should be utilized to address increases in psychological distress during the pandemic. Ongoing evaluation of the impact of lifestyle changes associated with the pandemic is needed.

## 1. Introduction

First reported in November 2019, the novel coronavirus (COVID-19) has resulted in a global health emergency. As of 3 June 2020, the virus has claimed more than 375,000 lives globally and infected more than 6.2 million people. The scale of the pandemic has resulted in worldwide concern, not only for the loss of life but also the social and economic impacts. There is significant concern over how the changes in the ways that people normally engage in everyday activities impact their health and well-being. This is especially relevant for those in self-isolation or quarantine, where feelings of depression, fear, guilt, and anger may manifest [1].

In Australia, similar to other countries, social distancing, travel bans, the cancellation of sporting and other mass participation events, and changes to work practices have dramatically affected daily life. The partial lockdown procedures implemented by the Australian government to protect citizens and reduce the spread of the virus forced the closure of many businesses in late March 2020 saw unemployment levels rise to 16.8%, more than three times higher than the pre-COVID-19 predicted unemployment rate [2]. The impact of these changes likely comes at significant personal cost, including the onset, or worsening, of mental health issues. To address the psychological distress experienced by Australians in the current pandemic, more than AUD74 million has been committed to the development and delivery of mental health and well-being support services in Australia. Multiple calls to ensure the preparedness of psychological services have been presented [3,4,5]; however, the uptake and immediate and long-term impacts of these services are unclear.

The COVID-19 pandemic may also lead to adverse changes in health behaviors, such as physical activity, smoking, alcohol use and sleep. With the enactment of social isolation and physical distancing restrictions in March 2020, the usual places to be physically active, such as gyms and outdoor recreation facilities, were no longer accessible. Although some people may have sufficient autonomous regulation of physical activity to pursue alternate activities (e.g., online fitness classes, other home-based physical activities), others may reduce their physical activity due to the lack of social support available or concerns for contracting the virus in an outdoor environment. On the other hand, those forced to work from home may have spent less time commuting, and may have seized the opportunity to create new physical activity habits. Alternatively, since exercise was one of few legitimate reasons for being able to leave the home some people may have developed a walking or cycling habit as a reason to escape being housebound. As many studies have demonstrated strong positive associations between physical activity and lower psychological distress [6,7], the commencement or continuation of physical activity during the pandemic will likely aid in reducing psychological distress. However, some concern has been expressed regarding increased risk of respiratory illness in those engaged in high- and very-high intensity exercise due to the potential for reduced immune response [8].

In contrast to health-promoting behavior such as physical activity, some people may manage social isolation and any pandemic-related psychological distress by commencing or increasing adverse health behaviors such as smoking or alcohol use. Since COVID-19 is an acute respiratory illness, commencement or continuation of tobacco use during the COVID-19 pandemic may lead to the worsening of outcomes for those infected with the virus [9]. Indeed, early indications suggest the proportion of current and former smokers is higher among those with severe disease and among those admitted to intensive care and requiring ventilation [10,11]. Harmful intake of alcohol leads to neuroadaptations that exacerbate alcohol cravings during times of stress [12]. Hence, social isolation, coupled with changes in employment status or uncertainty about the future may trigger an increase in alcohol intake for susceptible individuals [13]. 

The combined effect of changes in lifestyle behaviors; confinement to the home through government restrictions in travel; and elevated depression, anxiety and stress associated with the current COVID-19 pandemic, may have significant negative impacts on sleep [14]. This has been especially evident in healthcare workers, who may be required to work longer shifts in highly stressful environments [15,16]. Poorer sleep quality has been associated with higher levels of depression, stress, and anxiety [17]. Maintaining sleep quality is important in strengthening immunity [18], hence any sleep disturbances subsequent to COVID-19-pandemic-induced stress, may increase susceptibility to infection, or compromise recovery in the case of infection [19]. 

There is currently limited research regarding psychological distress subsequent to the COVID-19 pandemic. Two recent studies from China reported high levels of psychological distress during the initial stages of the pandemic [20,21]; however, the association between psychological distress and health behaviors remains unclear. Early evidence during the COVID-19 outbreak suggests positive associations between increased physical activity and physical health and inverse associations between sedentary behavior and physical and mental health outcomes [22]. A more detailed exploration of health behaviors during stages of the COVID-19 pandemic may help direct future public health messaging to promote positive behaviors and guard against uptake or the worsening of negative behaviors in order to maintain community well-being and mental health. Therefore, the present study aims to examine associations between depression, anxiety and stress and changes in health behaviors, including physical activity, sleep, smoking and alcohol use subsequent to the onset of COVID-19 and the implementation of social isolation rules in Australia. 

## 2. Materials and Methods

### 2.1. Participants and Recruitment

An anonymous online survey was hosted on the survey platform Qualtrics and distributed using social media sources (Facebook and Twitter) and via institutional sources including email and public marketing. Eligible participants included all Australian adults aged 18 years and over. Ethical approval was granted by Central Queensland University’s Human Research Ethics Committee (Approval number 22332). Data collection occurred between 9 and 19 April 2020. 

At the time of survey distribution, Australia was in the midst of significant personal distancing, partial lockdown and travel restrictions. Social distancing measures included keeping a minimum 1.5 meters between people, a ban on any public gatherings, a limit of no more than five people at personal gatherings such as weddings and funerals, and no person was allowed to meet with more than one other person outside of their own household. Lockdown restrictions also included the closure of restaurants and bars, many retail stores, and restricted access to outdoor parks. Most schools were closed, with students advised to study from home while being supported by online learning platforms and materials. University campuses limited or ceased face-to-face teaching and transitioned to online learning, with most clinical placements, residential schools, and simulations postponed. Inter- and intra-state travel was banned, and travel within towns and cities was only permitted for essential work/workers, or to access essential services such as medical or health care, or to shop for groceries. 

### 2.2. Survey Development

Existing COVID-19 surveys from China, the United Kingdom and Germany were reviewed to inform development of the present survey. In addition to demographic information, the survey included questions examining chronic health conditions; depression, anxiety and stress; and physical and health behaviors such as physical activity, sleep, smoking and alcohol consumption. The current paper only reports on measures included in the survey associated with the study aim. 

Demographic characteristics included age, gender, marital status, educational attainment, income and chronic disease status. Psychological distress was assessed using the well-established 21-item Depression, Anxiety and Stress Scale (DASS 21) [23]. Seven items for each component were scored on a 4-point Likert scale ranging from 0 (did not apply to me at all) to 3 (applied to me very much, or most of the time). Scores for depression, anxiety and stress items were summed with valid scores ranging from 0–21 for each component. Symptom severity was scored according standard cut-points [23]. 

Physical activity was assessed using the Active Australia Survey (AAS) which comprises eight items assessing frequency and duration of walking, moderate and vigorous leisure physical activities, and vigorous gardening over the past seven days. Total physical activity was calculated according to the AAS guidelines, where total minutes of physical activity = minutes of walking + minutes of moderate activity + (minutes of vigorous activity x 2) [24]. A single item asked participants to report their change in physical activity since the onset of COVID-19, with six response options ranging from 1 (I am much more physically active than usual) to 6 (I have ceased physical activity altogether).

Sleep was assessed using two items. First, participants were asked how many hours, on average, they slept per night prior to the onset of the COVID-19 pandemic (sleep quantity). Second, participants indicated the effect of the COVID-19 pandemic on current sleep quality using the question, “Since the onset of the COVID-19 pandemic, I...”. Five response options ranged from “am sleeping much better than usual” to “am sleeping much worse than usual”. 

Smoking behavior was assessed by asking whether respondents consumed cigarettes or other tobacco products prior to the onset of COVID-19. Change in smoking behavior was examined using a single item with ten response options ranging from “Since the onset of the COVID-19 pandemic, I... smoke much more than usual”, to “have not smoked (I am a non-smoker)”.

Current alcohol use was examined using the first item of the Alcohol Use Disorder Identification Test Consumption (AUDIT-C) [25], which asks how often alcohol is consumed. Response options were “Never”, “Monthly or less”, “2–4 times a month”, “2–3 times a week”, and “4 or more times a week”. Changes in alcohol consumption was assessed using a single self-report question: “Since the onset of the COVID-19 pandemic I…”, with the following response options: “drink much more than usual”, “drink a little more than usual”, “drink about the same as usual”, “drink less than usual”, “drink much less than usual”, “intend to reduce my drinking”, “intend to cease drinking”, or “have ceased drinking altogether”.

### 2.3. Statistical Analysis

SAS v9.4 (SAS Institute Inc., Lane Cove, Australia) was used for the analysis. The descriptive statistics, including frequencies and percentages, were generated for categorical variables; means and standard deviations (SD) were generated for continuous variables. Depression, anxiety and stress scores were compared based on participant’s sociodemographic and health status using non-parametric analysis of Wilcoxon rank-sum, the Kruskal–Wallis test and Spearman’s correlation. The responses for each behavior, i.e., physical activity, sleep, smoking and alcohol use, were recoded into negative change (−1), no change (0), or positive change (+1) for separate analyses of changes in each behavior. A multiple lifestyle behavior index [26] was created by summing the scores of the four behavior change items to reflect a composite health behavior change score, ranged from -4 to +4. The average composite health behavior change scores and SD were presented separately for each level of depression, anxiety and stress. Linear regression was used to test associations between composite health behavior change score and depression, anxiety and stress. Crude estimates and estimates adjusted for age, years of education, gender, marital status, household income and chronic disease status were reported with 95% Confidence Intervals (CI). Logistic regression was used to test whether negative changes in individual behavior change items were associated with depression, anxiety and stress. Crude odds ratios (OR) (Model 1) and ORs adjusted for age, years of education, gender, marital status, household income and chronic disease status (Model 2) with 95% CI were reported. All *p*-values were two-sided and considered significant if less than 0.05. 

## 3. Results

The sociodemographic and health characteristics of the study sample are presented in Table 1. In total, 1491 people (mean age 50.5 ± 14.9 years, 999 female) completed the survey. Most (*n* = 918, 62.8%) were married or in a relationship, and almost half (*n* = 693, 46.5%) reported having at least one chronic health condition. The average score for depression was 4.6 ± 5.0; anxiety, 2.2 ± 3.4; and stress, 5.2 ± 4.8. The average physical activity of participants was 312.5 minutes/week, but almost half (*n* = 729, 48.9%) reported a reduction in physical activity since the onset of the COVID-19 pandemic. The average sleep duration reported prior to the onset of COVID-19 was 7.1 ± 1.3 h per night, with half (*n* = 756, 50.7%) reporting no change in sleep quality since COVID-19. Most (*n* = 1319, 88.5%) were non-smokers, and the majority (*n* = 1228, 89.7%) reported no change in smoking since the onset of COVID-19. Almost one-quarter (*n* = 332, 22.3%) reported consuming alcohol on four or more occasions per week, and just over half (*n* = 825, 55.3%) reported no change in alcohol consumption. 

The depression, anxiety and stress scores in relation to different sociodemographic and health characteristics are presented in Table 2. No significant differences were found between males and females for depression and anxiety; however, females had significantly higher stress scores compared to males. Younger individuals (18–45 years) had significantly higher depression, anxiety and stress scores compared to their older counterparts. Similarly, those who were not in a relationship had significantly higher depression, anxiety and stress scores compared to other categories of relationship status. The Spearman’s correlation showed a significant negative association between years of education (recorded as a continuous variable) and scores for depression, but not for the anxiety or stress scores. Those in the lowest income category had significantly higher depression scores compared to higher income categories; however, no difference was observed between different weekly household incomes and anxiety and stress. Respondents who had been diagnosed with a chronic illness reported significantly higher depression, anxiety and stress scores, compared to those without chronic illness.

The mean changes in composite health behavior score, stratified by depression, anxiety and stress severity, are presented in Table 3. For depression, anxiety and stress, the number of people in each symptom severity category decreased as the symptom severity increased, except for the categories of extremely severe depression and anxiety. For depression, anxiety and stress, the mean composite health behavior change score decreased as the symptom severity increased, except for the categories of extremely severe anxiety and stress.

Associations between depression, anxiety and stress severity and negative change in behavior are outlined in Table 4. Since adjustment for age, years of education, gender, marital status, household income and chronic disease status did not impact associations, only adjusted OR’s are presented. Participants who reported a negative change in physical activity were more likely to have higher depression (adjusted OR = 1.08, 95% CI = 1.06, 1.11), anxiety (adjusted OR = 1.09, 95% CI = 1.05, 1.13), and stress (adjusted OR = 1.08, 95% CI=1.05, 1.11) symptoms. Those who reported a negative change in sleep were more likely to have higher depression (adjusted OR = 1.19, 95% CI = 1.15, 1.23), anxiety (adjusted OR = 1.25, 95% CI = 1.19, 1.31), and stress (adjusted OR = 1.30, 95% CI = 1.26, 1.35) symptoms. For those who reported a negative change in smoking, they were more likely to have higher depression (adjusted OR = 1.09, 95% CI = 1.04, 1.13), anxiety (adjusted OR = 1.12, 95% CI = 1.06, 1.18), and stress (adjusted OR = 1.10, 95% CI = 1.05, 1.15) symptoms. Similarly, those who reported a negative change in alcohol intake were more likely to have higher depression (adjusted OR = 1.07, 95% CI = 1.04, 1.10), anxiety (adjusted OR = 1.08, 95% CI = 1.04, 1.12), and stress (adjusted OR = 1.10, 95% CI = 1.07, 1.13) symptoms. The results were consistent for composite change scores. There was a decrease of 0.09 (95% CI = −0.10, −0.07), 0.10 (95% CI = −0.12, −0.07), and 0.10 (95% CI = −0.12, −0.08) points in composite change score for every point increase in depression, anxiety and stress.

## 4. Discussion

The present study examined the association between depression, anxiety and stress and the change in health behaviors of physical activity, sleep, smoking and alcohol use subsequent to the onset of COVID-19, as individual health behaviors and as a health behavior change index composite score. The main findings were that all aspects of psychological distress (depression, anxiety and stress) were significantly associated with changes in health behavior, both independently and as a composite score. Numerous studies have examined the association between a range of health behaviors and psychological distress factors. For example, Rebar and colleagues reported significant inverse associations between physical activity participation and depression and anxiety levels in their meta-analysis [27]. Previous work has reported significant positive associations between smoking, and depression [28], but not between smoking cessation and reductions in depression or anxiety [29]. Large-scale studies also demonstrate a significant association between alcohol misuse and psychological distress [30]. Taken together, the findings of previous work suggest variability in the associations between lifestyle behaviors and depression, anxiety and stress that appear to depend on the nature of the behavior under investigation.

The present study also demonstrated that, as the severity of depression increased, the composite health behavior change score worsened. That is, those with normal levels of depression symptoms reported a small negative change (−0.42 points), while for those with extremely severe symptoms, the change in composite health behavior change score was more than three times greater (−1.45). For anxiety and stress, as symptom severity increased from normal to severe, so did negative changes in composite health behavior change score. Linear regression showed a significant association between increased depression, anxiety and stress, and negative changes in composite health behavior change scores. Logistic regression showed that, compared to no change or positive change, a negative change in all behaviors was associated with a significantly greater likelihood of increased depression, anxiety and stress. 

A number of reports suggest COVID-19 is likely to have significant impacts on psychological distress [21,31]; however, the data from the present study suggest that the mean scores for depression, anxiety and stress are mostly within the normal to mild range. Moreover, the mean scores for depression and stress were only slightly elevated when compared to normative data for Australian adults, and anxiety the scores were marginally lower [32]. Viewed another way, more than 60% of all respondents reported psychological distress within the normal range, and less than 13% reported severe to extremely severe scores. The mean scores for depression, anxiety and stress in the present study are all substantially lower than those reported in Italy. Mazza and colleagues [33] reported mean depression, anxiety and stress scores of 5.34, 2.89, and 7.43, respectively, compared to 4.6, 2.2, and 5.2, respectively, in the present study. These differences may be accounted for in the timing of data collection as data from Italy were collected in mid-March, differences in government responses to the pandemic, and differences in the severity of impact on the population.

The prevalence of moderate to severe depression in the present study (19.1%) is comparable to that reported in China (16.8%) [21]; however, the prevalence of moderate to severe anxiety is markedly less in this study (8.3%) compared to that in China (28.8%). In contrast, the prevalence of moderate to severe stress reported in this study (15.1%) is almost double that reported in China (8.1%). The timing of data collection may account for some of these differences since the data from China were collected from residents in 194 cities during late January–early February, one day after the World Health Organization declared a public health emergency. In contrast, the data for the present study were collected in early to mid-April when significant travel and social distancing restrictions were already in place. It is possible that the low prevalence of depression may also be a result of government investment in mental health support services. The lower anxiety scores in the present study may be attributed to respondents being somewhat accustomed to changes in social contact, whereas the higher stress levels may be attributed to the uncertainly about the future, particularly regarding job losses and economic stress.

The total average physical activity was 322.5 ± 36.5 min/week. This is similar to recent Australian Bureau of Statistics data based on the Active Australia Survey, showing that Australians aged 15 and over reported 42 min of daily activity, or 294 min per week on average [34], but substantially less than the peak of 541 min of activity per week reported by Alley and colleagues using the same measure [35]. Physical activity guidelines for Australian adults suggest they should accumulate 150–300 min of moderate intensity physical activity, 75–150 min of vigorous intensity physical activity, or an equivalent combination of both, per week. However, here we report total physical activity, not moderate or moderate-to-vigorous activity. The AAS is known to overreport physical activity participation, but actigraphy is not practical in large samples [36]. Therefore, our data may be an over-representation of actual physical activity performed.

Almost half of our respondents (48.9%) reported a negative change (reduction) in physical activity since the onset of the COVID-19 pandemic, but about 20% also reported a positive change. This is important to note, since there has been considerable emphasis in the media on the importance of maintaining physical activity for physical and mental health benefits [37,38]. Our data suggests these recommendations may have been ineffective for most people, but not all. These data are hard to interpret as there has been a visible increase in people using walking paths all over the country, as well as a strong increase in registrations to the 10,000 Steps Australia program [39]. It may be that the extra people who are walking are predominantly those who were already active (e.g., gym and sports club members) prior to the COVID-19 onset, but had to undertake different activities at different locations due to the closure of exercise and sporting facilities. The reported overall decline in physical activity is likely a consequence of social distancing, travel restrictions, the closure of usual exercise venues, or unwillingness to change previous exercise habits. Nonetheless, given the psychological distress responses to COVID-19, [40] and the established benefits of physical activity on psychological distress [41,42], additional strategies to promote physical activity are needed. 

Prior to the COVID-19 pandemic, mean sleep duration was 7.1 ± 1.3 h, which meets the guidelines of 7–9 h for adults [43] and aligns with a recent national study of Australian adults [44]. Although half (50.7%) of all respondents reported no change in sleep quality since the onset of the COVID-19 pandemic, 40.7% reported a negative change. This is unsurprising given the potential for psychological distress during a global pandemic, change in exercise behaviors, and employment and relationship concerns. A number of recommendations have been made to address poor sleep during COVID-19, including maintaining a regular sleep routine, taking time for self-reflection, limiting exposure to COVID-19-related news, and getting regular exercise during daylight hours [14]. Apart from these COVID-19-specific recommendations, most principles mirror those recommended for good sleep hygiene in usual circumstances. 

Only 11% of survey respondents were smokers. This is less than the 15% prevalence of smoking recently reported among Australian adults [45]. Overwhelmingly, respondents have not changed their smoking behavior, with almost 93% reporting no change or a positive change (reduction) in smoking status since the onset of the COVID-19 pandemic. Among smokers, 16.3% (*n* = 28) report a positive change (reduction), 38.4% (*n* = 59) report no change, while 49.9% (*n* = 85) report a negative change (increase) in smoking behavior. Since COVID-19 is a respiratory illness, and smokers are more susceptible to respiratory tract infections, there is significant potential for adverse events in this population. Early evidence from China suggests either a significant association, [46] or at least a trend [47] toward smoking being associated with poor prognosis in COVID-19 cases. To date, there has been limited attention in the media to smoking cessation programs or adverse risk associated with smoking. Although more research is needed, health promotion efforts directed at educating the population regarding the risks for smokers during the COVID-19 pandemic are needed. These may include higher exposure to passive smoking during periods of lockdown or relapse-preventions strategies targeting those who have recently ceased smoking.

Almost three quarters of respondents reported no change or a positive change (reduction) in alcohol use since the onset of COVID-19. A reduction in alcohol use might be driven by closures to licensed establishments such as bars and clubs and temporary restrictions on alcohol purchases. In contrast, around one quarter of respondents reported a negative change (increase) in alcohol consumption. This is consistent with research by Australia’s Foundation for Alcohol Research and Education [48] reporting that 20% of Australians increased alcohol purchases since the onset of COVID-19 and 70% were drinking more than usual. Worryingly, this report suggests that almost 30% of adults are drinking more to cope with psychological distress [48]. Concerns such as these have prompted the Australian Government to invest more than AUD6 million into drug- and alcohol-related services to combat the risk of substance abuse and related harms, such as domestic violence, due to the pandemic.

To the best of our knowledge, this is the first published study to report associations between health behaviors and psychological distress in Australian adults during the COVID-19 pandemic. One published study from Europe reported that reductions in physical activity and increased sedentary behaviors during lockdown were associated with negative changes in physical and mental health [22]. Moreover, a number of reports have highlighted the need for rapid and comprehensive responses to increasing mental health needs during COVID-19 [3,5]; however, it is expected this support will need to be maintained for some years to come given the magnitude of the COVID-19 pandemic.

There are a number of strengths of the present study, including the inclusion of multiple health behaviors, a large sample size, and the timing of data collection relative to lockdown restrictions in Australia. However, there are also some limitations to consider. Firstly, all data are self-reported meaning responses are subject to recall bias. Secondly, data are cross-sectional and therefore causality cannot be inferred. Thirdly, participants in the present study are older compared to other studies examining health behaviors such as sleep [49], and thus the generalizability to other populations needs to be confirmed. Additionally, longitudinal data are needed to observe changes over time to assess the impact of changes in social restrictions. Finally, our sample was recruited conveniently and therefore the results may not be generalizable to populations with different characteristics. 

## 5. Conclusions

In conclusion, our data suggests negative changes in health behaviors are associated with increased psychological distress in Australian adults during the COVID-19 pandemic. Effective health promotion strategies directed at adopting or maintain positive health-related behaviors such as targeted social media messaging and balanced media reporting, should be used to reduce the acute and chronic increases in psychological distress during these unprecedented times. Ongoing evaluation of the impact of lockdown rules and social distancing (associated with the pandemic) on health behaviors is necessary to inform these targeted health promotion strategies.

## Figures and Tables

**Table 1:** Sample characteristics.

	*N*	Percentage or Mean (SD)
Age (years)	1491	50.5 (14.9)
**Gender**		
Male	484	32.6
Female	999	67.4
**Marital status**		
Never married	300	20.5
Divorced/Separate/Widowed	243	16.6
Married/In a relationship	918	62.8
Years of Education	1491	16.3 (5.1)
**Household income**		
<$1000/week	335	26.1
$1000–<$2000/week	381	29.7
≥$2000/week	568	44.2
**Chronic disease status**		
No	798	53.5
Yes	693	46.5
**DASS score**		
Depression	1491	4.6 (5.0)
Anxiety	1491	2.2 (3.4)
Stress	1491	5.2 (4.8)
**Physical activity (mins/week)**	1491	312.5 (363.5)
**Sleep (hrs/night)**	612	7.1 (1.3)
**Smoker**		
Yes	172	11.5
No	1319	88.5
**Alcohol consumption**		
Never	301	20.2
Monthly or less	322	21.6
2–4 times per week	250	16.8
2–3 times per week	286	19.2
4 or more times per week	332	22.3
**Change in physical activity**		
Negative change	729	48.9
No change	454	30.5
Positive change	308	20.7
**Change in sleep quality**		
Negative change	607	40.7
No change	756	50.7
Positive change	128	8.6
**Change in smoking**		
Negative change	103	6.9
No change	1338	89.7
Positive change	50	3.4
**Change in alcohol**		
Negative change	396	26.6
No change	825	55.3
Positive change	270	18.1

**Table 2:** Difference in psychological distress based on sociodemographic and health characteristics.

Characteristic	Depression Mean (SD)	*p*-Value	Anxiety Mean (SD)	*p*-Value	Stress Mean (SD)	*p*-Value
**Gender**						
Female	4.5 (4.8)	0.189	2.2 (3.2)	0.108	5.3 (4.7)	0.005 *
Male	4.6 (5.4)		2.2 (3.7)		4.9 (5.1)	
**Age (years)**						
18–45	6.0 (5.5)	<0.001 *	3.0 (3.9)	<0.001 *	7.1 (5.1)	<0.001 *
46 to 65	4.0 (4.7)		2.0 (3.2)		4.6 (4.5)	
>65	3.0 (3.9)		1.2 (2.2)		2.5 (3.3)	
**Marital status**						
Never married	6.7 (5.9)	<0.001 *	3.5 (4.6)	<0.001 *	6.9 (5.4)	<0.001 *
Divorced/Separated/Widowed	5.2 (5.6)		2.3 (3.5)		4.5 (4.8)	
Married/In a relationship	3.8 (4.2)		1.8 (2.8)		4.8 (4.5)	
**Years of education** (continuous variable) ^^^	−0.096	0.002 *	−0.074	0.318	−0.017	0.555
**Household income (AUD)**						
<$1000/week	5.2 (5.7)	0.047 *	2.6 (3.7)	0.106	4.9 (5.0)	0.132
$1000–<$2000/week	4.7 (5.1)		2.3 (3.3)		5.2 (4.8)	
≥$2000/week	4.1 (4.6)		2.0 (3.1)		5.3 (4.7)	
**Diagnosed with a chronic disease**						
No	4.3 (4.9)	0.001 *	1.8 (3.0)	<0.0001 *	4.9 (4.8)	0.003 *
Yes	5.0 (5.2)		2.7 (3.7)		5.5 (4.9)	

* Denotes significant difference between categories; ^ Denotes *p* value of Spearman’s correlation between years of education and depression score; AUD—Australian Dollar.

**Table 3:** Composite health behavior change score stratified by depression, anxiety and stress severity.

Psychological Distress Factor Severity (Range of Scores)	*n* (%)	Composite Health Behavior Change Score Mean (SD)
**Depression**		
Normal (0–4)	920 (61.7%)	−0.42 (1.25)
Mild (5–6)	175 (11.7%)	−1.03 (1.25)
Moderate (7–10)	206 (13.8%)	−1.22 (1.34)
Severe (11–13)	79 (5.3%)	−1.28 (1.42)
Extremely severe (>13)	111 (7.4%)	−1.45 (1.44)
**Anxiety**		
Normal (0–3)	1175 (78.8%)	−0.58 (1.28)
Mild (4–5)	115 (7.7%)	−1.20 (1.40)
Moderate (6–7)	83 (5.6%)	−1.24 (1.35)
Severe (8–9)	40 (2.7%)	−1.50 (1.52)
Extremely severe (>9)	78 (5.2%)	−1.23 (1.61)
**Stress**		
Normal (0–7)	1077 (72.2%)	−0.49 (1.25)
Mild (8–9)	143 (9.6%)	−1.34 (1.23)
Moderate (10–12)	121 (8.1%)	−1.25 (1.46)
Severe (13–16)	105 (7.0%)	−1.43 (1.43)
Extremely severe (>16)	45 (3.0%)	−1.33 (1.69)

**Table 4:** Associations between psychological distress and negative change in health behavior.

Adjusted Model (*n* = 1256) ^#^			
	Depression	Anxiety	Stress
**Logistic Regression–Odds Ratio (95% CI)**			
Physical activity	1.08 * (1.06, 1.11)	1.09 * (1.05, 1.13)	1.08 * (1.05, 1.11)
Sleep	1.19 * (1.15, 1.23)	1.25 * (1.19, 1.31)	1.30 * (1.26, 1.35)
Smoking	1.09 * (1.04, 1.13)	1.12 * (1.06, 1.18)	1.10 * (1.05, 1.15)
Alcohol drinking	1.07 * (1.04, 1.10)	1.08 * (1.04, 1.12)	1.10 * (1.07, 1.13)
**Linear Regression–score estimate (95% CI)**			
Composite change score	−0.09 * (−0.10, −0.07)	−0.10 * (−0.12, −0.07)	−0.10 * (−0.12, −0.08)

Reference for logistic regression is “no change/positive change”. * *p* < 0.001; ^#^ Adjusted for age, years of education, gender, marital status, household income and chronic disease status.
